# Supplementary material for: Enhancement of an In Vivo Anti-Inflammatory Activity of Oleanolic Acid through Glycosylation Occurring Naturally in Stauntonia hexaphylla
Source: Molecules. 2020 Aug 13;25(16):3699. doi: 10.3390/molecules25163699 (PMC7464308; doi:10.3390/molecules25163699)
Supplement: Supplementary file 1 [file molecules-25-03699-s001.pdf]

Supplementary material

Enhancement of an In Vivo Anti-Inflammatory Activity of Oleanolic Acid through Glycosylation Occurring Naturally in *Stauntonia hexaphylla*

Le Ba Vinh [1,2,†], Nguyen Thi Minh Nguyet [1,†], Liu Ye [1], Gao Dan [1], Nguyen Viet Phong [2], Hoang Le Tuan Anh [3], Young Ho Kim [1], Jong Seong Kang [1], Seo Young Yang [1,*] and Inkyu Hwang [1,*]

[1] College of Pharmacy, Chungnam National University, Daejeon 34134, Korea; vinhrooney@gmail.com (L.B.V.); minhnguyet.usth@gmail.com (N.T.M.N.); liuye1995@naver.com (L.Y.); gaodan521361@hotmail.com (G.D.); yhk@cnu.ac.kr (Y.H.K.); kangjss@cnu.ac.kr (J.S.K.)

[2] Institute of Marine Biochemistry (IMBC), Vietnam Academy of Science and Technology (VAST), Hanoi 100000, Vietnam; ngvietphong@gmail.com

[3] Mientrung Institute for Scientific Research, Vietnam Academy of Science and Technology (VAST), Thua Thien Hue 531600, Vietnam; hoangletuananh@hotmail.com

* Correspondence: syyang@cnu.ac.kr (S.Y.Y.); hwanginkyu@cnu.ac.kr (I.H.); Tel.: +82-42-821-7321 (S.Y.Y); +82-42-821-5922(I.H.)

† These authors contributed equally to this work.

Abstract: *Stauntonia hexaphylla* (Lardizabalaceae) has been used as a traditional herbal medicine in Korea and China for its anti-inflammatory and analgesic properties. As part of a bioprospecting program aimed at the discovery of new bioactive compounds from Korean medicinal plants, a phytochemical study of *S. hexaphylla* leaves was carried out leading to isolation of two oleanane-type triterpene saponins, 3-*O*-[*β*-D-glucopyranosyl (1→2)-*α*-L-arabinopyranosyl] oleanolic acid-28-*O*-[*β*-D-glucopyranosyl (1→6)-*β*-D-glucopyranosyl] ester (**1**) and 3-*O*-*α*-L-arabinopyranosyl oleanolic acid-28-*O*-[*β*-D-glucopyranosyl (1→6)-*β*-D-glucopyranosyl] ester (**2**). Their structures were established unambiguously by spectroscopic methods such as one- and two-dimensional nuclear magnetic resonance and infrared spectroscopies, high-resolution electrospray ionization mass spectrometry and chemical reactions. Their anti-inflammatory activities were examined for the first time with an animal model for the macrophage-mediated inflammatory response as well as a cell-based assay using an established macrophage cell line (RAW 264.7) in vitro. Together, it was concluded that the saponin constituents, when they were orally administered, exerted much more potent activities in vivo than their sapogenin core even though both the saponins and the sapogenin molecule inhibited the RAW 264.7 cell activation comparably well in vitro. These results imply that saponins from *S. hexaphylla* leaves have a definite advantage in the development of oral medications for the control of inflammatory responses.

Keywords: anti-inflammatory effect; Lardizabalaceae; oleanane triterpene saponin; *Stauntonia hexaphylla*

CONTENTS **Page**

Acid hydrolysis and sugar identification 3

Figure S1. Certification of *S. hexaphylla* from National Institute of Biological Resources 4

Figure S2. Results of genetic analysis of the plant material *S. hexaphylla* 5

Figure S3. ^{1}H-NMR spectrum (C_5D_5N, 600 MHz) of compound **1** 5

Figure S4. ^{13}C-NMR spectrum (C_5D_5N, 150 MHz) of compound **1** 6

Figure S5. HMQC spectrum (C_5D_5N, 600 MHz) of compound **1** 6

Figure S6. HMBC spectrum (C_5D_5N, 600 MHz) of compound **1** 7

Figure S7. COSY spectrum (C_5D_5N, 600 MHz) of compound **1** 7

Figure S8. ^{1}H-NMR spectrum (C_5D_5N, 600 MHz) of compound **2** 8

Figure S9. ^{13}C-NMR spectrum (C_5D_5N, 150 MHz) of compound **2** 8

Figure S10. HMQC spectrum (C_5D_5N, 600 MHz) of compound **2** 9

Figure S11. HMBC spectrum (C_5D_5N, 600 MHz) of compound **2** 9

Figure S12. COSY spectrum (C_5D_5N, 600 MHz) of compound **2** 10

Figure S13. The HPLC-ELSD chromatogram of EtOH extract of *S. hexaphylla* leaves and standard mixture of compounds **1** and **2** 10

Figure S14. The HPLC-UV chromatogram of EtOH extract of *S. hexaphylla* leaves and standard mixture of compounds **1** and **2** determined at 210 nm. 11

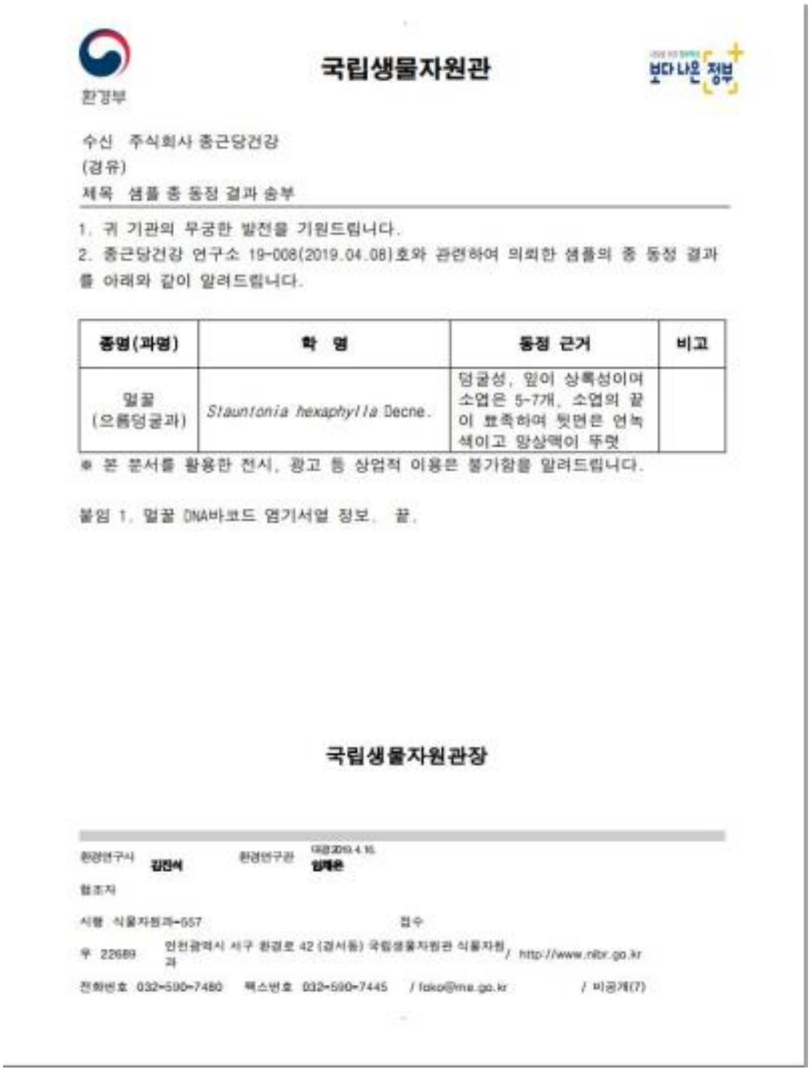

환경부

국립생물자원관

보다 나은 정부

수신 주식회사 종근당건강
(경유)
제목 샘플 종 동정 결과 송부

1. 귀 기관의 무궁한 발전을 기원드립니다.
2. 종근당건강 연구소 19-008(2019.04.08)호와 관련하여 의뢰한 샘플의 종 동정 결과를 아래와 같이 알려드립니다.

종명(과명)	학 명	동정 근거	비고
멀꿀 (으름덩굴과)	*Stauntonia hexaphylla* Decne.	덩굴성, 잎이 상록성이며 소엽은 5-7개, 소엽의 끝이 뾰족하며 뒷면은 연녹색이고 망상맥이 뚜렷	

※ 본 문서를 활용한 전시, 광고 등 상업적 이용은 불가함을 알려드립니다.

붙임 1. 멀꿀 DNA바코드 염기서열 정보. 끝.

국립생물자원관장

환경연구사 김진석 환경연구관 대결 2019.4.16. 임채은
협조자
시행 식물자원과-557 접수
우 22689 인천광역시 서구 환경로 42 (경서동) 국립생물자원관 식물자원과 / http://www.nibr.go.kr
전화번호 032-590-7480 팩스번호 032-590-7445 / lokoi@me.go.kr / 비공개(7)

Figure S1. Certification from National Institute of Biological Resources (Ministry of Environment, Korea) of *Stauntonia hexaphylla*

World Meridian Center 10F, 254 Beotkkot-ro, Geumcheon-gu, Seoul, Republic of Korea
Tel: 82-2-2180-7261 Fax: 02-2180-7100 Email: info@macrogen.com

macrogen

Sample Name

Sample Name	sample6_ITS

Analysis Report

Name	Read Length (Normal)	Read Length (Q16)	Read Length (Q20)	GC Content
**sample6_ITS**	**761**	**719**	**719**	**63.73193166885677**
sample6_ITS_R	729	725	724	64.0603566529492 5
sample6_ITS_F	722	718	715	64.40443213296399

Contig Sequence

GGTGTACAAGGTTTCCGTAGGTGAACCTGCGGAAGGATCATTGTCGAAACCTGCCCCGACAGGACCACCCGTGAATCGTCGATGCAAAATCGTTCG
GGGAGGGGCGTCGCGCGAGCGGCGATCCCCCCTCCCGTCGTGCCGCGAGCCCTGCCCGGATCGTCGGCGCGGACCCTGTCCGCGCCGCGACGCCCC
GCCGTCCTCGTGGTCTCGAATTAACAACGTATCCCCGGCGCGATCAGCGTCAAGGAAATTTCACAAGACGCGCGTCTCCGTGGCCCGCCCGGCTTT
GCGGTGCGTCCACGGACGGCGTCGCGATCCTGATATTTGAACGACTCTCGGCAACGGATATCTCGGCTCTCGCATCGATGAAGAACGTAGCGAAAT
GCGATACTTGGTGTGAATTGCAGAATCCCGTGAACCATCGAGTTTTTGAACGCAAGTTGCGCCCTAGGCCGTTAGGTCGAGGGCACGTCTGCCTGG
GCGTCAAGCATCGCGTCACCCCCGACTCCGTCGTCTCACCGCGAGATCCCCCGGAGCCGCGTGGGTGGAGATTGGCCCCCCGTGCGTCGCGCGCGG
TCGGCCCAAAAACGAGCCCCCGACGGCCGGTGTCACGATCAGTGGTGGTTGACGTGCCTCTTTCCGAAGATGGATGTCGTGCCCGCCGCGTCGTCG
AACGGGCCATCCGGACCTTGTTGGTGCTCACGGAGCACTCGTCCTGCGACCCCAGGTCAGGCGGGGCCACCCGCTGAGTTTAAGCATAT

BlastN Report

Query		Subject					Score			Identities		
Start	End	Description	AC	Leng	Start	End	Bit	Raw	EV	Matc	Total	Pct.(%)
44	724	Stauntonia hexaphylla internal transcribed spacer 1, 5.8S ribosomal RNA gene, and internal transcribed spacer 2, complete sequence	AY029784.1	681	1	680	1251	677	0.0	680	681	99
[illegible]	725	[illegible] internal transcribed spacer 1, 5.8S ribosomal RNA gene, and internal transcribed spacer 2, complete sequence	AY029793.1	678	1	678	1162	629	0.0	665	682	98
44	725	Parvatia brunoniana subsp. elliptica internal transcribed spacer 1, 5.8S ribosomal RNA gene, and internal transcribed spacer 2, complete sequence	AY029781.1	680	1	680	1160	628	0.0	665	683	97
44	725	Holboellia angustifolia subsp. angustifolia internal transcribed spacer 1, 5.8S ribosomal RNA gene, and internal transcribed spacer 2, complete sequence	AY029790.1	680	1	680	1158	627	0.0	664	682	97
44	721	Holboellia latifolia isolate 8A internal transcribed spacer 1, partial sequence; 5.8S ribosomal RNA gene, complete sequence; and internal transcribed spacer 2, partial sequence	MH270479.1	677	1	677	1157	626	0.0	661	678	97

Figure S2. Results of genetic analysis of *S. hexaphylla*

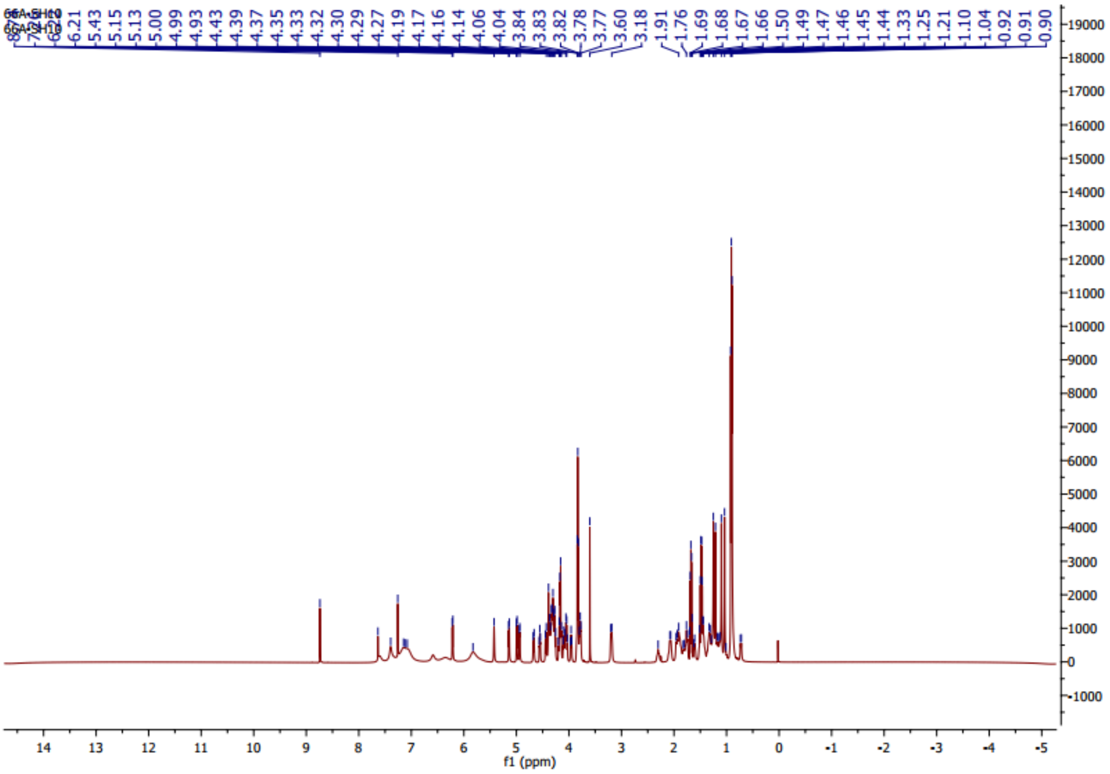

Figure S3. ^{1}H-NMR spectrum (Pyridine-d_5, 600 MHz) of compound **1**

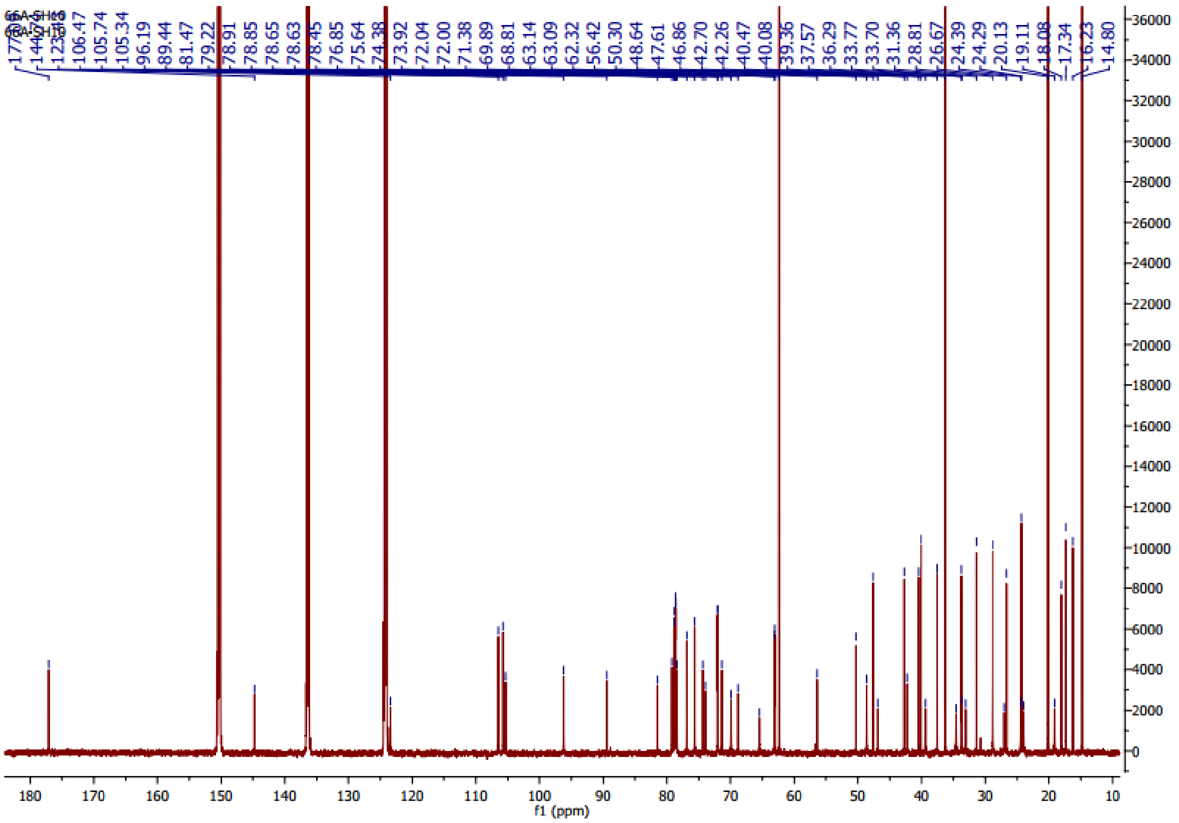

Figure S4. ^{13}C-NMR spectrum (Pyridine-d_5, 150 MHz) of compound **1**

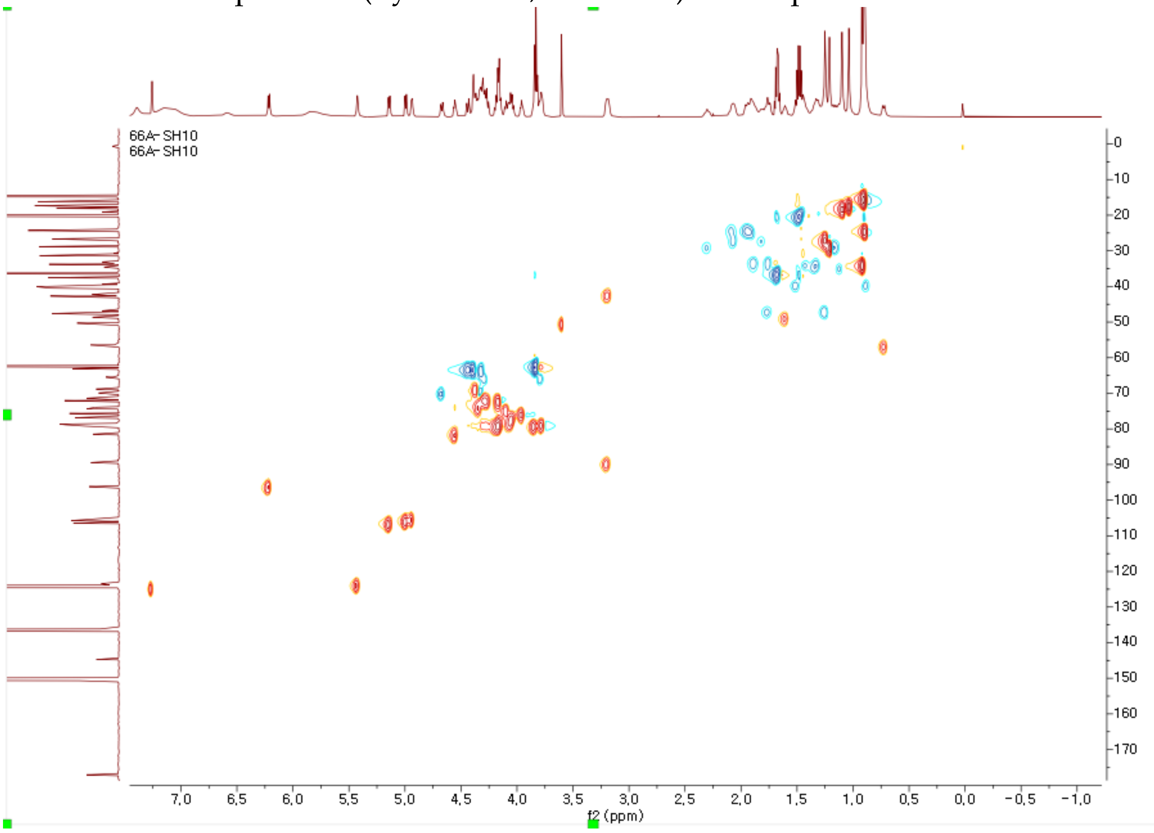

Figure S5. HSQC spectrum (Pyridine-d_5, 600 MHz) of compound **1**

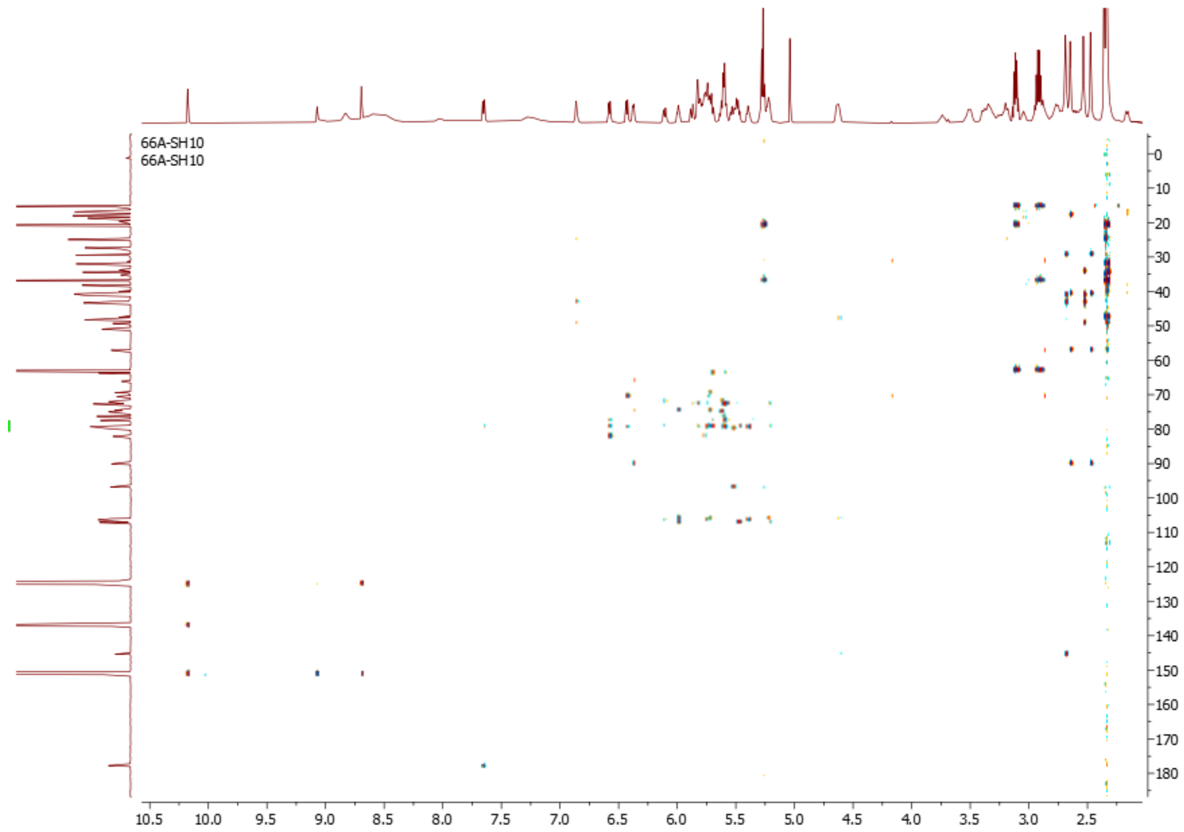

Figure S6. HMBC spectrum (Pyridine-*d*5, 600 MHz) of compound **1**

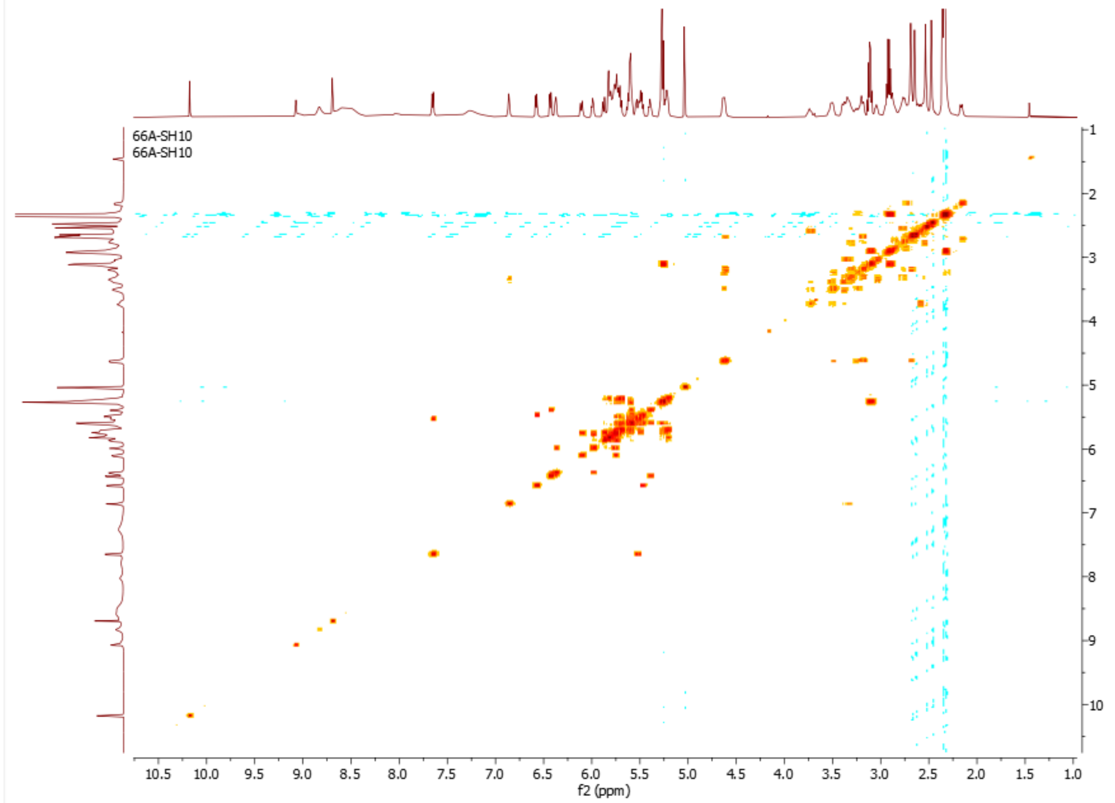

Figure S7. COSY spectrum (Pyridine-*d*5, 600 MHz) of compound **1**

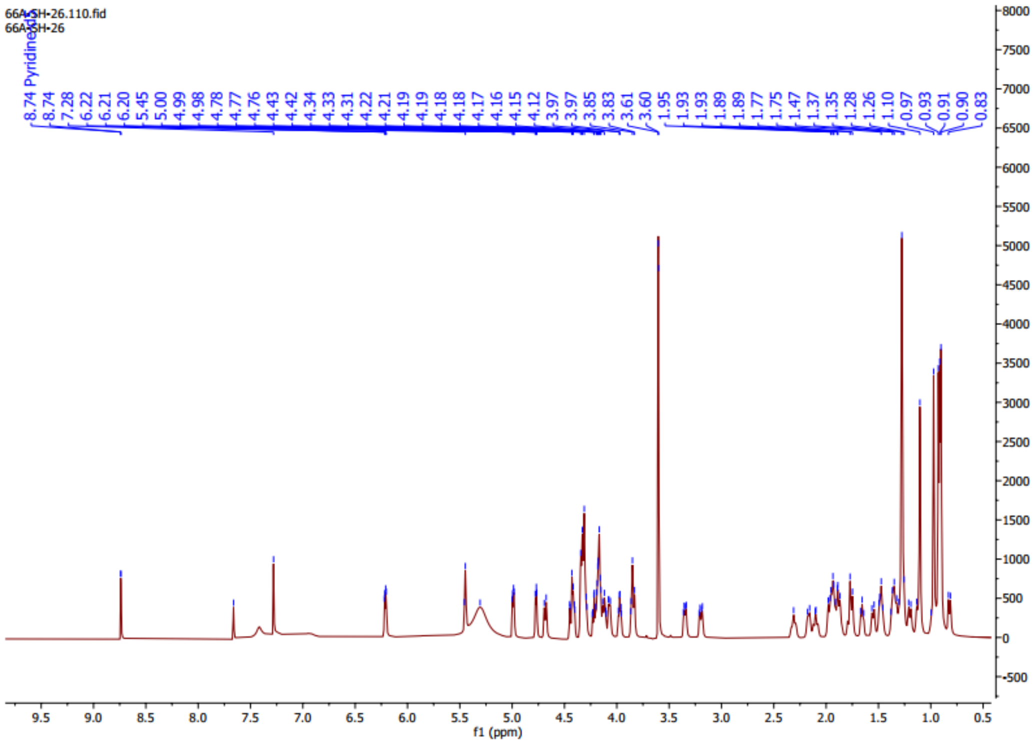

Figure S8. ^{1}H-NMR spectrum (Pyridine-*d*$_5$, 600 MHz) of compound **2**

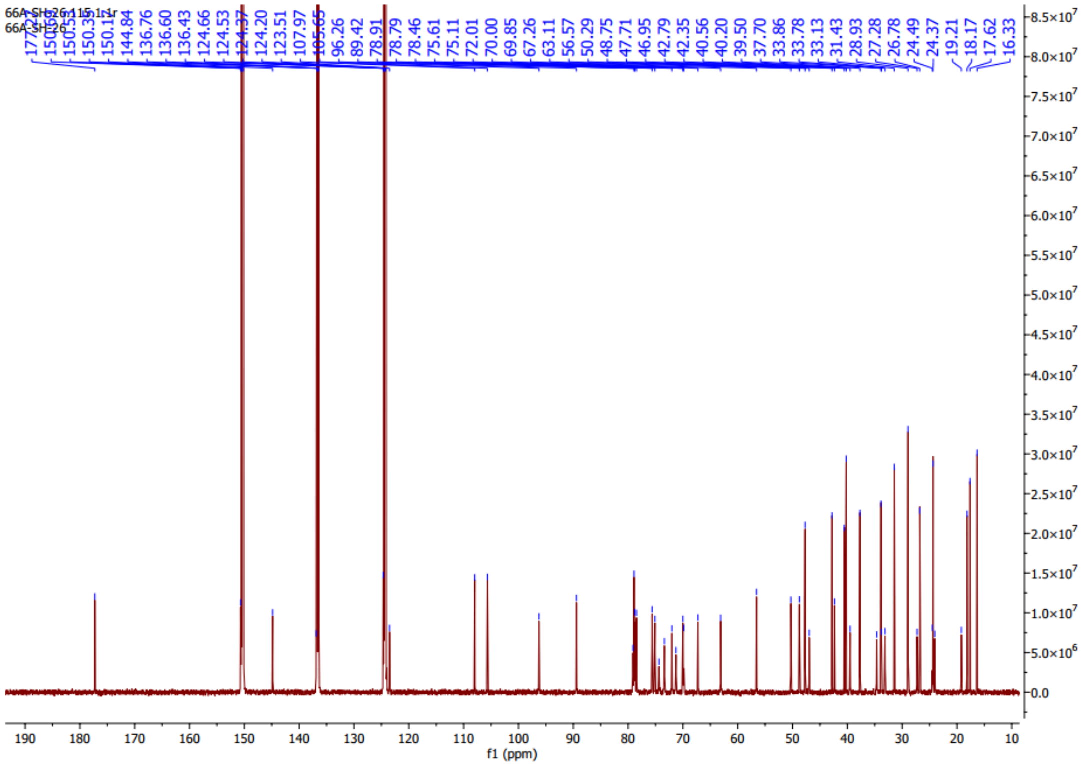

Figure S9. ^{13}C-NMR spectrum (Pyridine-*d*$_5$, 150 MHz) of compound **2**

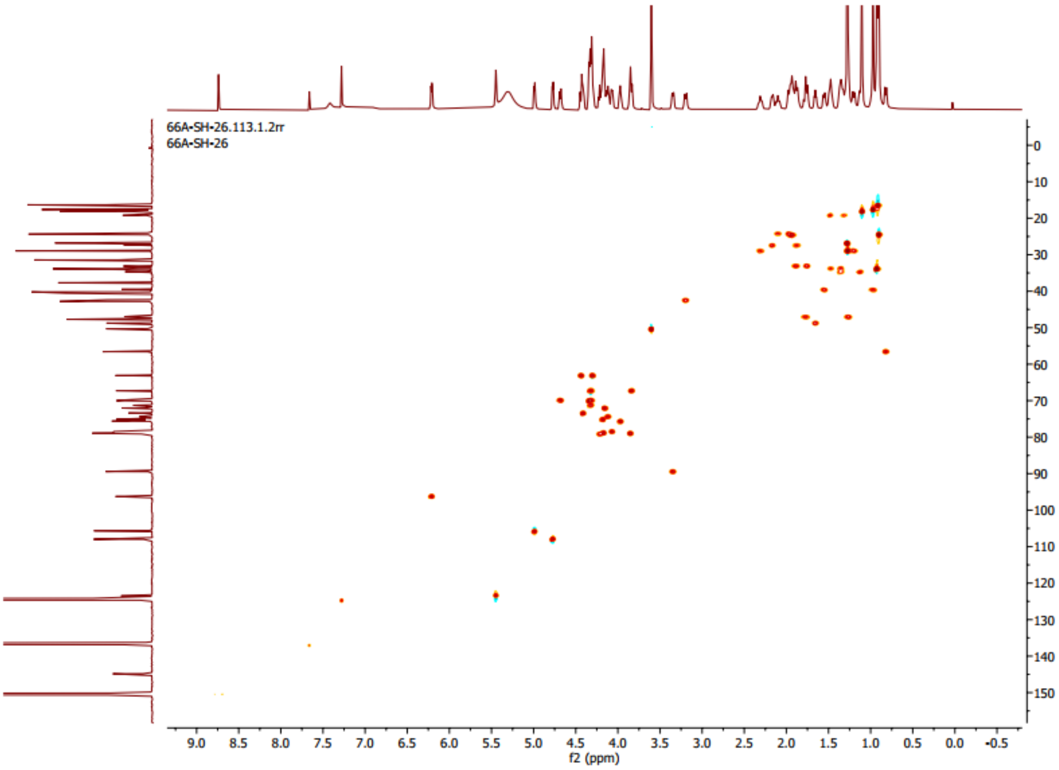

Figure S10. HSQC spectrum (Pyridine-d_5, 600 MHz) of compound **2**

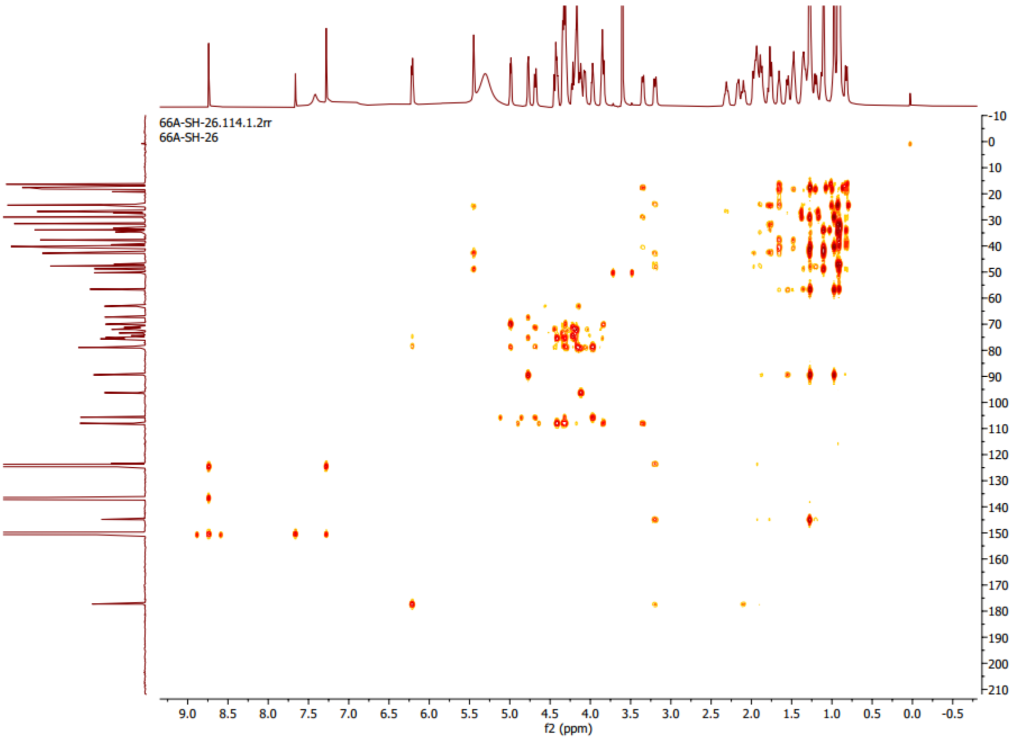

Figure S11. HMBC spectrum (Pyridine-d_5, 600 MHz) of compound **2**

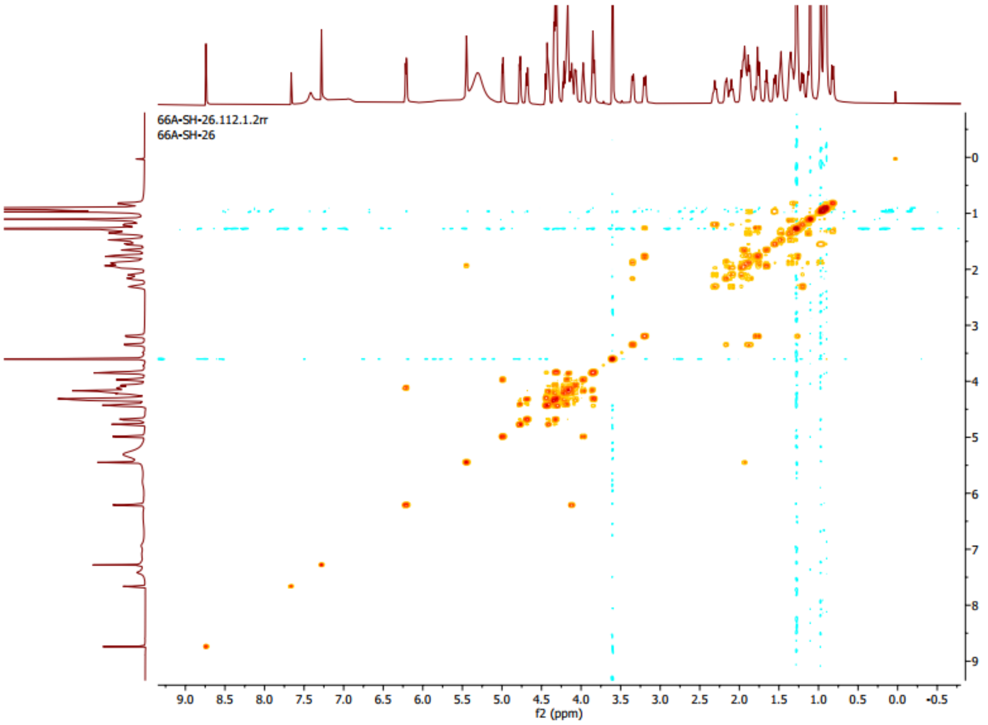

Figure S12. COSY spectrum (Pyridine-d_5, 600 MHz) of compound **2**

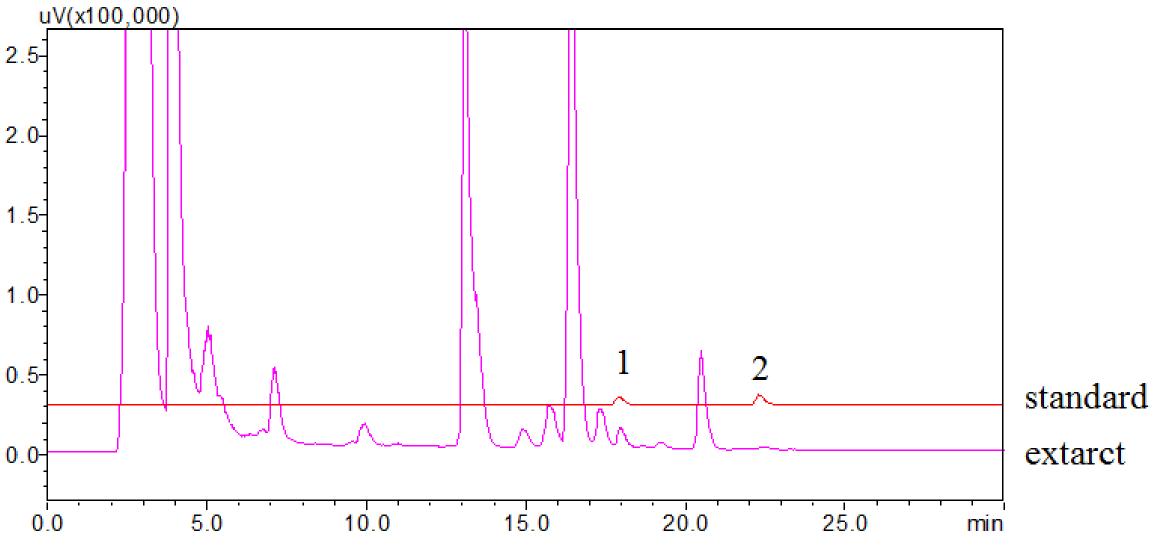

Figure S13. The HPLC-ELSD chromatogram of EtOH extract of *S. hexaphylla* leaves and standard mixture of compounds **1** and **2**.

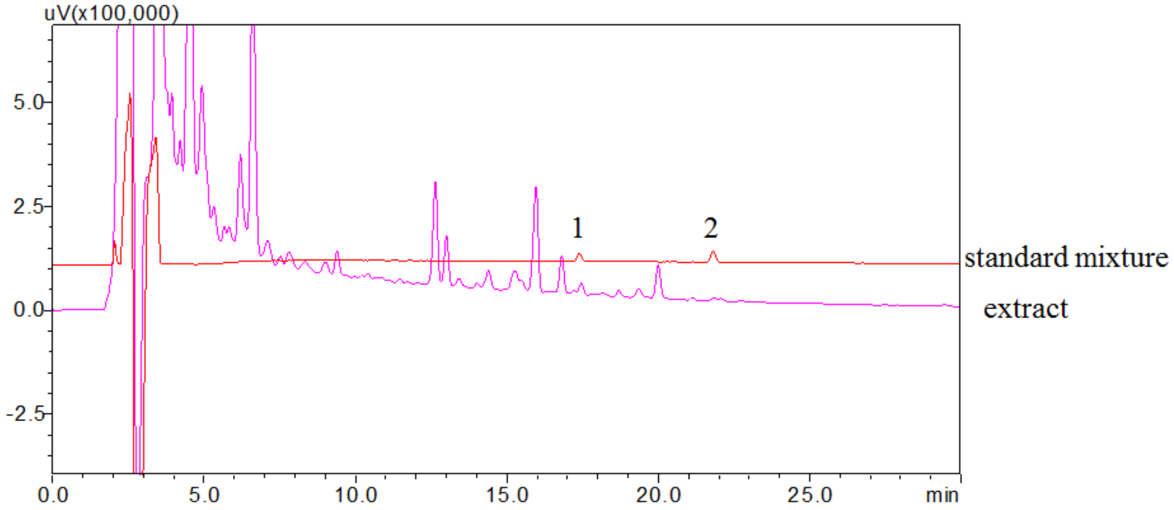

Figure S14. The HPLC-UV chromatogram of EtOH extract and standard mixture of compounds **1** and **2** determined at 210 nm.
